# Proton Beam Therapy for Pediatric Tumors of the Central Nervous System—Experiences of Clinical Outcome and Feasibility from the KiProReg Study

**DOI:** 10.3390/cancers14235863

**Published:** 2022-11-28

**Authors:** Sarah Peters, Sabine Frisch, Annika Stock, Julien Merta, Christian Bäumer, Christoph Blase, Eicke Schuermann, Stephan Tippelt, Brigitte Bison, Michael Frühwald, Stefan Rutkowski, Gudrun Fleischhack, Beate Timmermann

**Affiliations:** 1 West German Proton Therapy Center Essen (WPE), University Hospital Essen, 45147 Essen, Germany; 2 Clinic for Particle Therapy, University Hospital Essen, 45147 Essen, Germany; 3 Department of Neuroradiology, University Hospital Wuerzburg, 97080 Wuerzburg, Germany; 4 AnästhesieNetz Rhein-Ruhr, Westenfelder Str. 62/64, 44867 Bochum, Germany; 5 Department of Pediatric Hematology and Oncology, Pediatrics III, University Hospital Essen, 45147 Essen, Germany; 6 Diagnostic and Interventional Neuroradiology, Faculty of Medicine, University of Augsburg, 86156 Augsburg, Germany; 7 Neuroradiological Reference Center for the Pediatric Brain Tumor (HIT) Studies of the German Society of Pediatric Oncology and Hematology, University Hospital Würzburg, 97080 Würzburg, Germany; 8 Pediatric and Adolescent Medicine, Swabian Childrens Cancer Center, University Medical Center Augsburg, 86156 Augsburg, Germany; 9 Department of Pediatric Hematology and Oncology, University Medical Center Hamburg-Eppendorf, 20251 Hamburg, Germany; 10 West German Cancer Center (WTZ), 45147 Essen, Germany; 11 German Cancer Consortium (DKTK), 45147 Essen, Germany

**Keywords:** proton beam therapy, childhood cancer, brain cancer, adverse events, imaging changes

## Abstract

**Simple Summary:**

Radiation therapy is an important cornerstone of the treatment of many different types of brain tumors occurring in childhood. Proton beam therapy offers the opportunity to reduce doses outside of the target volume due to its physical characteristics. By sparing a large volume of the brain from radiation doses, proton beam therapy aims at reducing long-term side effects and preserving cognitive function. Our study aims at better understanding side effects and therefore contributing to better treatment decisions in this vulnerable group of patients. Therefore, the study analyses outcome and side effects including imaging changes in a large cohort of children with brain tumors from a prospective registry.

**Abstract:**

As radiotherapy is an important part of the treatment in a variety of pediatric tumors of the central nervous system (CNS), proton beam therapy (PBT) plays an evolving role due to its potential benefits attributable to the unique dose distribution, with the possibility to deliver high doses to the target volume while sparing surrounding tissue. Children receiving PBT for an intracranial tumor between August 2013 and October 2017 were enrolled in the prospective registry study KiProReg. Patient’s clinical data including treatment, outcome, and follow-up were analyzed using descriptive statistics, Kaplan–Meier, and Cox regression analysis. Adverse events were scored according to the Common Terminology Criteria for Adverse Events (CTCAE) 4.0 before, during, and after PBT. Written reports of follow-up imaging were screened for newly emerged evidence of imaging changes, according to a list of predefined keywords for the first 14 months after PBT. Two hundred and ninety-four patients were enrolled in this study. The 3-year overall survival of the whole cohort was 82.7%, 3-year progression-free survival was 67.3%, and 3-year local control was 79.5%. Seventeen patients developed grade 3 adverse events of the CNS during long-term follow-up (new adverse event *n* = 7; deterioration *n* = 10). Two patients developed vision loss (CTCAE 4°). This analysis demonstrates good general outcomes after PBT.

## 1. Introduction

Pediatric tumors of the central nervous system (CNS) predominantly occur at a young age [1]. Due to improving treatment opportunities, life expectancy has increased for many of the affected patients. Therefore, reducing long-term side effects and preserving cognitive function are utterly important for this very vulnerable group of patients. As radiation therapy (RT) is an important part of the treatment in a variety of pediatric tumors of the CNS, tolerability of RT in children has been discussed with concern since its introduction [2]. Several studies reported possible impairment of neurological function after RT in children dependent on dose and irradiated volume [3,4,5,6]. Therefore, proton beam therapy (PBT) plays an evolving role because of its potential benefits to deliver high doses to the target volume while sparing surrounding tissue [7,8,9]. Over time, more data have evolved, supporting low toxicity after PBT [10,11,12,13]. Even in very young children, recent studies have demonstrated good feasibility [14].

Treatment of children with CNS malignancies regularly involves other treatment options such as surgery and chemotherapy (CTX) prior to RT. Apart from tumor-specific treatments, some children require additional interventions such as a ventriculo-peritoneal shunt (VP shunt), with implanted medical devices bringing a risk of malfunction or infection potentially leading to subsequent interventions. In addition, disease itself can lead to neurocognitive symptoms. All of these factors may influence the condition of the child before RT and need to be taken into account for treatment planning as well as dose prescription [15]. Possibilities for dose reductions need to be considered, but are limited in order not to compromise the aim of high survival outcomes [16].

Today, there is an increasing interest in transient changes on magnetic resonance imaging (MRI) following RT. However, information on incidence and clinical relevance is sparse and different classification systems for post-therapeutic imaging events are proposed, either based on imaging or on clinical findings [17,18,19,20,21].

The primary aim of this study was to determine outcome and feasibility of PBT in patients enrolled in a large, prospective registry study. Survival data for the whole cohort as well as for several subgroups were evaluated. Longitudinal analysis was conducted on neurotoxicity data. Furthermore, this study aimed to evaluate clinical risk factors for side effects, in order to improve decisions for treatment planning for these patients. In addition, the frequency of imaging changes after PBT in pediatric patients was investigated in a retrospective analysis of the cohort.

## 2. Materials and Methods

For this study, patients under the age of 18 years, treated with PBT for an intracranial tumor of the CNS at the West German Proton Therapy Center Essen until October 2017 with a minimal follow-up (FU) time of 6 months were examined. Patients had to be enrolled in the prospective registry study (KiProReg; German Clinical Trials Register: DRKS00005363) after formal consent of their legal guardian(s). Approval for this study has been granted by the local ethical committee. Patients with spinal location of their primary tumor and patients without any available FU after PBT were excluded.

Methods of treatment and data collection have been previously published [15]. Face-to-face FU consultations with a radiation oncologist were scheduled after three months and annually thereafter at our center. If an FU visit was not possible in person, appointments were held via phone or via a questionnaire. In addition, letters of referring physicians were evaluated. FU information was collected before initiation of PBT, weekly during PBT, and at every aftercare appointment. Adverse events were classified according to CTCAE Version 4.0. CTCAE scores before start of PBT were considered as baseline. Maximum CNS CTCAE scores at baseline were compared with the scores at the last available FU within three years. We analyzed changes from baseline to last FU and considered them as stable, deterioration, or improvement. During FU, imaging was performed by the referring hospital according to their standards and respective treatment protocols. In general, the first MRI after PBT took place within three months and thereafter every three to six months. In order to assess imaging events, the following MRI sequences were recommended: T1-weighted images (T1WI), T2-weighted images (T2WI), diffusion-weighted images (DWI), susceptibility-weighted images (SWI), T2 fluid-attenuated inversion recovery sequence (FLAIR), and T1WI after contrast media application. MRI scans and written reports were required as part of the aftercare. In a retrospective analysis, written reports for the first 14 months after PBT were screened for any evidence of imaging changes, according to keywords that were defined together with the reference radiologist from the German pediatric brain tumor network (Table 1). Characteristics of patients meeting criteria for imaging changes were analyzed in a subgroup.

IBM SPSS Statistics version 24.0 (IBM Corp. in Armonk, NY, USA) and R statistic software version 4.0.2 (R Foundation for Statistical Computing, Vienna, Austria) was used for data management and statistical calculations. We assessed distribution and relationship of attributes and compared them, using cross tables and a chi-square test. Survival data were defined as the time from the end of PBT to any event. An event for overall survival (OS) was defined by death of any cause. For the progression-free survival (PFS), an event was defined as evidence of any disease progression and for local control (LC) as evidence of local progression or recurrence. In the absence of an event, the date of last FU was used for censoring OS. Dates of death or last FU were used for PFS and LC, respectively. Survival rates were estimated using the Kaplan–Meier method. Differences were tested using the log rank test. Statistical significance was defined as a *p* value ≤ 0.05. A multivariable Cox regression was performed to evaluate prognostic factors for OS, PFS, and LC. Included variables were gender, age above four years, dose over 59 Gy (RBE), use of anesthesia, CTX concomitant to PBT, craniospinal irradiation (CSI), metastatic disease, extension of prior tumor surgery, and treatment for salvage. Variable selection was guided by backward stepwise selection using the elimination criterion of a *p* value > 0.1. For the four most frequent entities, additional subgroup analysis was performed.

## 3. Results

### 3.1. Patient Characteristics

**Figure 1:**
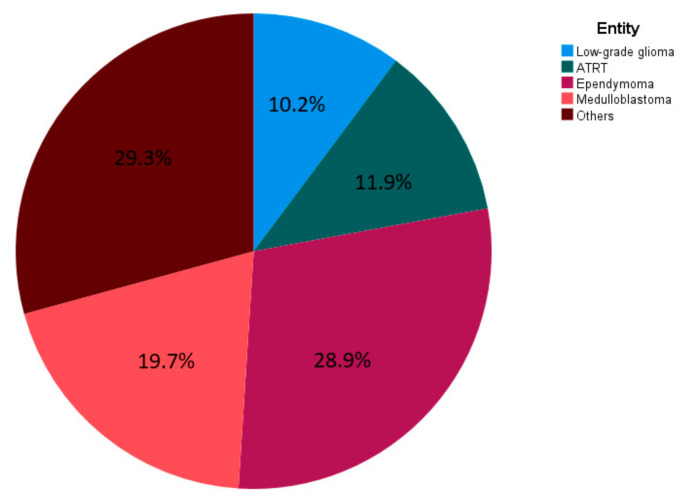
Entities of CNS tumors among included patients.

Two hundred and ninety-four patients met the inclusion criteria for this study. Four patients had to be excluded due to missing FU. Patients with 18 entities were included. The most frequent ones were ependymomas and medulloblastomas (Figure 1). Details on histopathological diagnoses are listed in Table A1 of the Appendix A.

Patient characteristics are described in Table 2. The majority of patients were male. At initiation of PBT, 37.6% of the patients were younger than four years. Forty-one patients (13.9%) had metastatic disease before PBT. A fossa posterior syndrome prior to PBT was diagnosed in 21 patients (7.2%). In 26 patients (8.8%), MRI reports suggested signs of apoplexy before initiation of PBT. Intrathecal CTX had been administered in 59 patients (20.1%) prior to PBT. Twenty-six patients (8.8%) received at least one course of high-dose chemotherapy prior to PBT. Two patients terminated treatment prematurely due to deterioration of general condition; one of them with a new diagnosis of an acute myeloid leukemia (AML) during the course of PBT. In 33.3% of the patients receiving concomitant CTX, dose of CTX had to be reduced in at least one course. Median number of concomitant substances was two (range, 0–5). After PBT, seven patients received at least one course of high-dose chemotherapy. Hospitalization for other reasons than CTX was necessary in 51 patients for a median of 6.5 days (range, 2–61 days).

Patient details grouped by entity are displayed in Table A2 of the Appendix A. 

### 3.2. Survival Data

One hundred and ninety-nine patients achieved disease control following PBT. Disease progression was observed in 95 patients and occurred either as local recurrence (*n* = 43), metastatic dissemination (*n* = 34), or both (*n* = 18). Median time from PBT to progression was 7.5 months (range, 0.1–76.1 months). Fifty-four patients died. Cause of death was disease progression in 50 cases. One patient died due to a rapid progression of an AML diagnosed during course of PBT. Another patient died due to multifactorial vascular causes. One patient died due to hemorrhage after surgery for stenosis of the internal carotid artery. One cause of death remained unknown. The estimated 3-year OS, PFS, and LC rates of the whole cohort were 82.7%, 67.3%, and 79.5%, respectively. On univariate analysis, metastatic disease and anesthesia were significant negative factors for OS, younger age and anesthesia for PFS, and higher dose, younger age, and anesthesia for LC, respectively. On multivariable Cox regression, anesthesia remained significant for all survival outcomes. Higher dose remained significant for LC and was also significant for PFS. All results of univariate and multivariate analysis of risk factors for survival are displayed in Table 3 and Table 4.

#### 3.2.1. Subgroup Ependymoma

**Figure 2:**
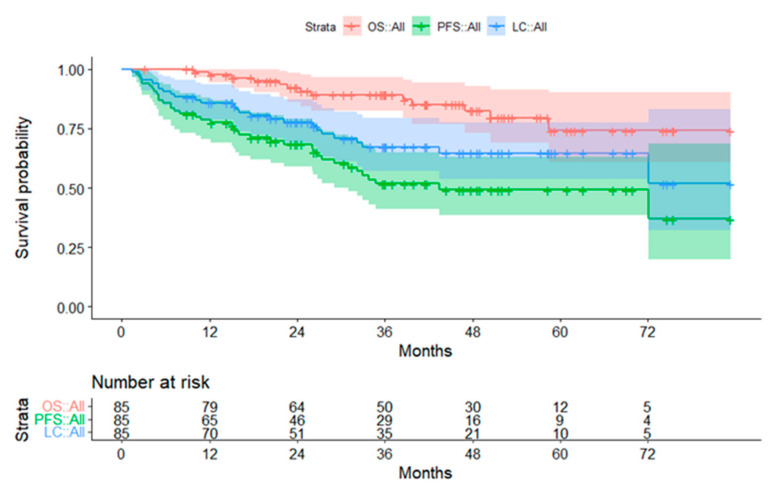
Survival curves for patients with ependymoma. The 95% confidence intervals are displayed. Abbreviations: OS = overall survival; PFS = progression-free survival; LC = local control.

In 38 out of 85 children with ependymoma, progression of disease occurred. The first progression was local in 21 patients, 12 developed metastases, and combined local and disseminated failure occurred in five. Median FU of children, who were still alive, was 41.7 months (range, 3.2–83.3 months). The 3-year OS, PFS, and LC were 89.2%, 51.8%, and 67.3%, respectively. In patients treated in the primary setting, the 3-year OS, PFS, and LC were 96.7%, 58.4%, and 70.8%, respectively (Figure 2). Compared to the group of patients treated at tumor recurrence or progression, the OS (*p* = 0.001) and PFS (*p* < 0.001) were significantly superior for patients treated at primary diagnosis. Considering only the non-pre-irradiated patients in the relapsed group, the 3-year OS, PFS, and LC were 85.7%, 35.6%, and 48.6%, respectively. Here, the difference to the primary group was no longer significant (OS *p* = 0.750; PFS *p* = 0.093; LC *p* = 0.161).

#### 3.2.2. Subgroup Medulloblastoma

**Figure 3:**
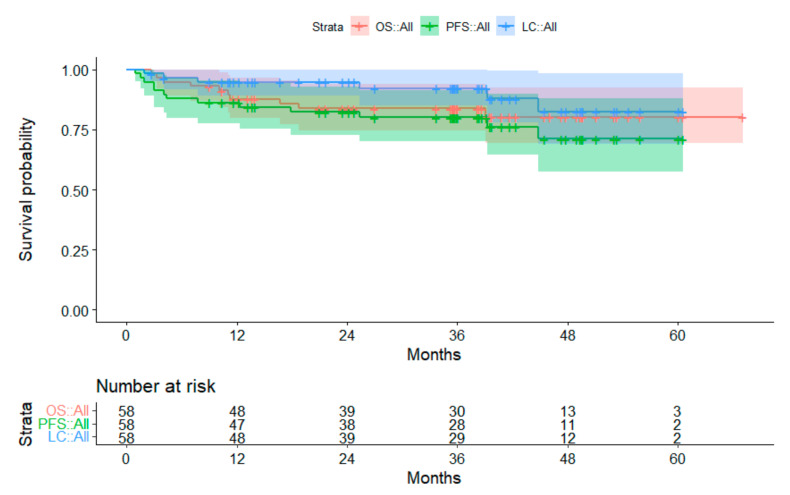
Survival curves for patients with medulloblastoma. The 95% confidence intervals are displayed. Abbreviations: OS = overall survival; PFS = progression-free survival; LC = local control.

Forty-five out of 58 patients with medulloblastoma achieved disease control. Thirteen patients experienced progression of disease. The first progression was local (*n* = 2), metastatic (*n* = 7), or both (*n* = 4). By the end of the evaluation, ten patients had died. Survivors had a median FU of 38.6 months (range, 9.0–67.1 months). The 3-year OS was 83.8%. The 3-year PFS and 3-year LC were 80.0% and 92.2%, respectively. For patients treated in the primary setting, 3-year OS, PFS, and LC were 89.1%, 84.2%, and 92.2%, respectively (Figure 3).

#### 3.2.3. Subgroup Atypical Teratoid Rhabodid Tumors

**Figure 4:**
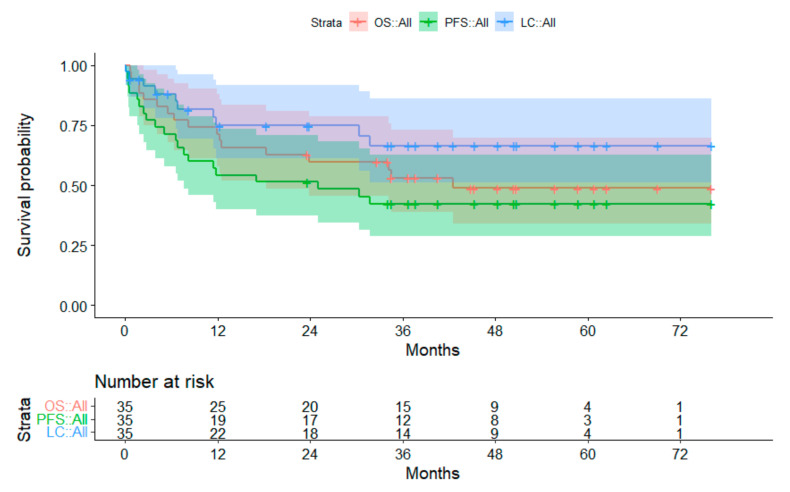
Survival curves for patients with atypical teratoid rhabdoid tumor. The 95% confidence intervals are displayed. Abbreviations: OS = overall survival; PFS = progression-free survival; LC = local control.

Progression of disease was observed in 20 out of 35 children with atypical teratoid rhabdoid tumor (ATRT) (57.1%). Initial progression was local (*n* = 6), metastatic (*n* = 10), or both (*n* = 4). Seventeen patients died after disease progression (initial sides were local = 5; metastatic = 9; both = 3). The 3-year OS was 53.2%. The 3-year-PFS and LC were 42.4% and 66.5%, respectively (Figure 4). Disease progression occurred between 0.1 and 31.8 months after PBT. FU of survivors was a median of 46.8 months (range, 23.6–76.1 months). Fourteen patients had an FU longer than 34 months without disease progression.

#### 3.2.4. Subgroup Low-Grade Glioma

**Figure 5:**
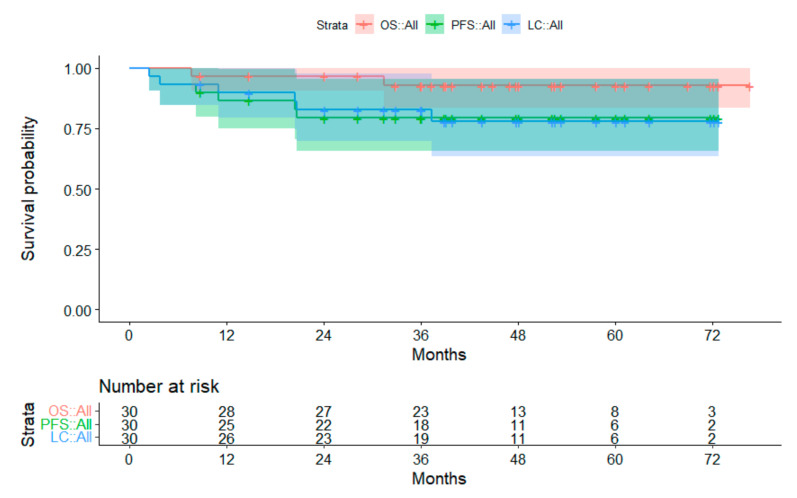
Survival curves for patients with low-grade glioma. The 95% confidence intervals are displayed. Abbreviations: OS = overall survival; PFS = progression-free survival; LC = local control.

Twenty-four out of 30 children with low-grade glioma did not experience disease progression. Two patients died, one from local tumor progression, the other one from hemorrhage after surgery. Eight children had local failure or local failure with metastasis. Median FU of the living patients was 47.4 months (range, 8.8–16.7 months). The 3-year OS was 92.8% for the overall cohort. The 3-year PFS and LC were 79.3% und 82.7%, respectively (Figure 5).

### 3.3. Adverse Events

Data regarding any CTCAE criteria were available for 231 patients three months after PBT, for 189 patients one year after PBT, for 147 patients two years after PBT, for 110 three years after PBT, and for 62 four years after PBT, respectively. Before starting PBT, half of the patients presented already with impairments of the CNS including 22 patients with grade 3 findings. Other high-grade toxicity (≥3) before initiating PBT concerned blood and the lymphatic system (*n* = 19), the skin and subcutaneous tissue (*n* = 1), musculoskeletal and connective tissue (*n* = 1), metabolism and nutrition (*n* = 6), hearing (*n* = 6), and the eye (*n* = 22). 

During the course of PBT, low-grade adverse events (grade 1–2) of the skin occurred in 268 patients. High-grade hematological toxicities were documented in 66 patients, mostly in association with concomitant CTX. Fever grade ≤ 2 occurred in 26 patients, nine of them received concomitant CTX (not significant (n.s.)). Six patients had longer interruptions of PBT of ≥six days due to malfunction of a VP shunt (*n* = 4) or infection of a Port-a-cath. Two patients had to terminate PBT prematurely; one due to a newly diagnosed AML and one due to suspected disease progression.

**Figure 6:**
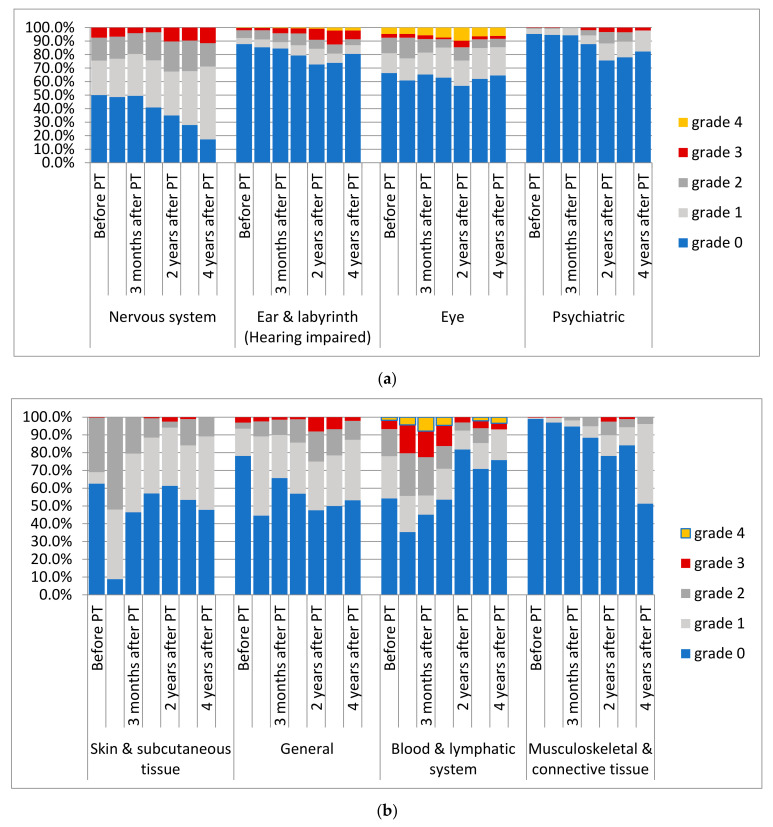
(**a**,**b**) Adverse events according to CTCAE Version 4.0, before PBT, during PBT, 3 months after PT, and 1, 2, 3, and 4 years after PBT.

With regard to late toxicities after PBT, two patients developed new vision loss scored grade 4. For these patients, Dmax and D50 of the chiasm were 55.42 Gy (RBE)/53.16 Gy (RBE) and 57.70 Gy (RBE)/51.07 Gy (RBE), respectively. High-grade ototoxicity (grade ≥ 3) was reported in 4.8% of the patients. One child developed hearing impairment grade 4 as a deterioration of a pre-existing condition. Thirteen children developed grade 3 ototoxicity (new *n* = 8; deterioration *n* = 5). Adverse events of grade 3 of the skin were reported in five patients’ questionnaires during long-term FU (skin atrophy *n* = 4; dry skin *n* = 1). In one of these children, skin atrophy was reported after a second RT. In 15 children, 17 general grade 3 adverse events were observed. Pre-existing gait disturbance and fatigue deteriorated in two patients, respectively. New reported adverse events were gait disturbance (*n* = 7), fatigue (*n* = 4), and pain (*n* = 2). Seven patients developed new grade 3 adverse events of the central nervous system during long-term follow-up. Three children presented with memory impairment and/or cognitive disturbance, one developed chronic headaches, two developed ataxia during tumor recurrence, and one patient experienced symptomatic radiation necrosis after two further RT courses at another institution. In ten patients, deterioration of pre-existing conditions resulted in a grade 3 toxicity. Further details of prevalence of adverse events of the whole cohort are presented in Figure 6a,b.

**Figure 7:**
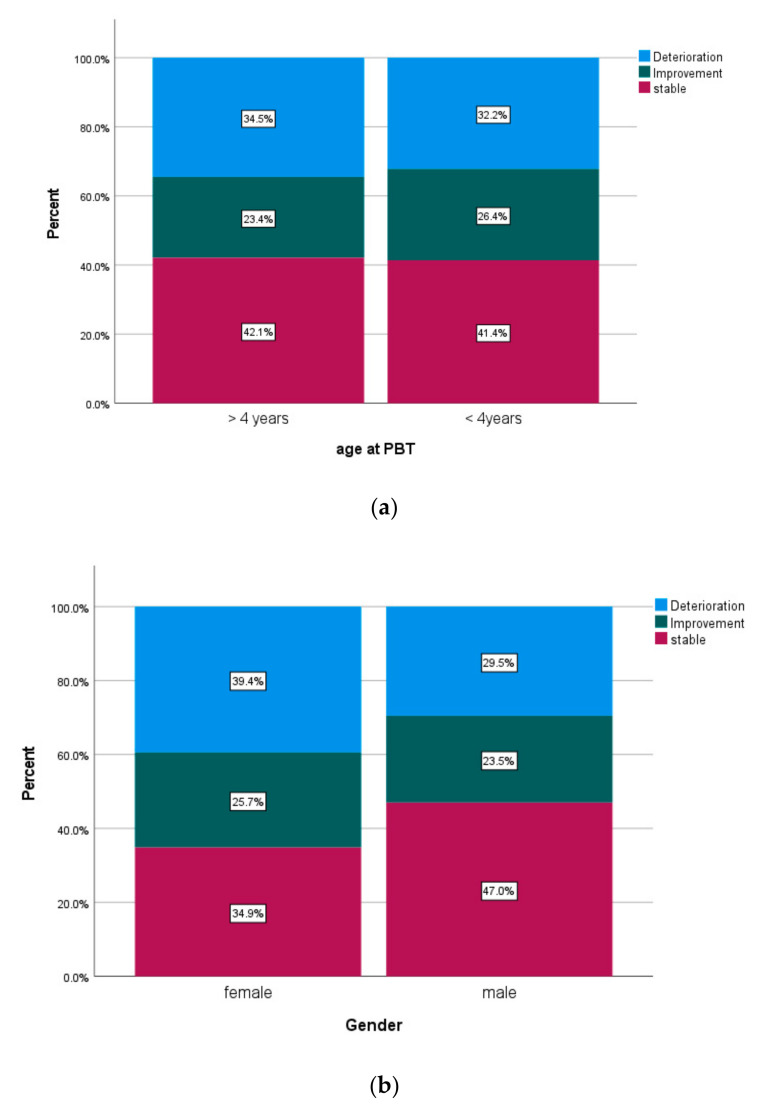

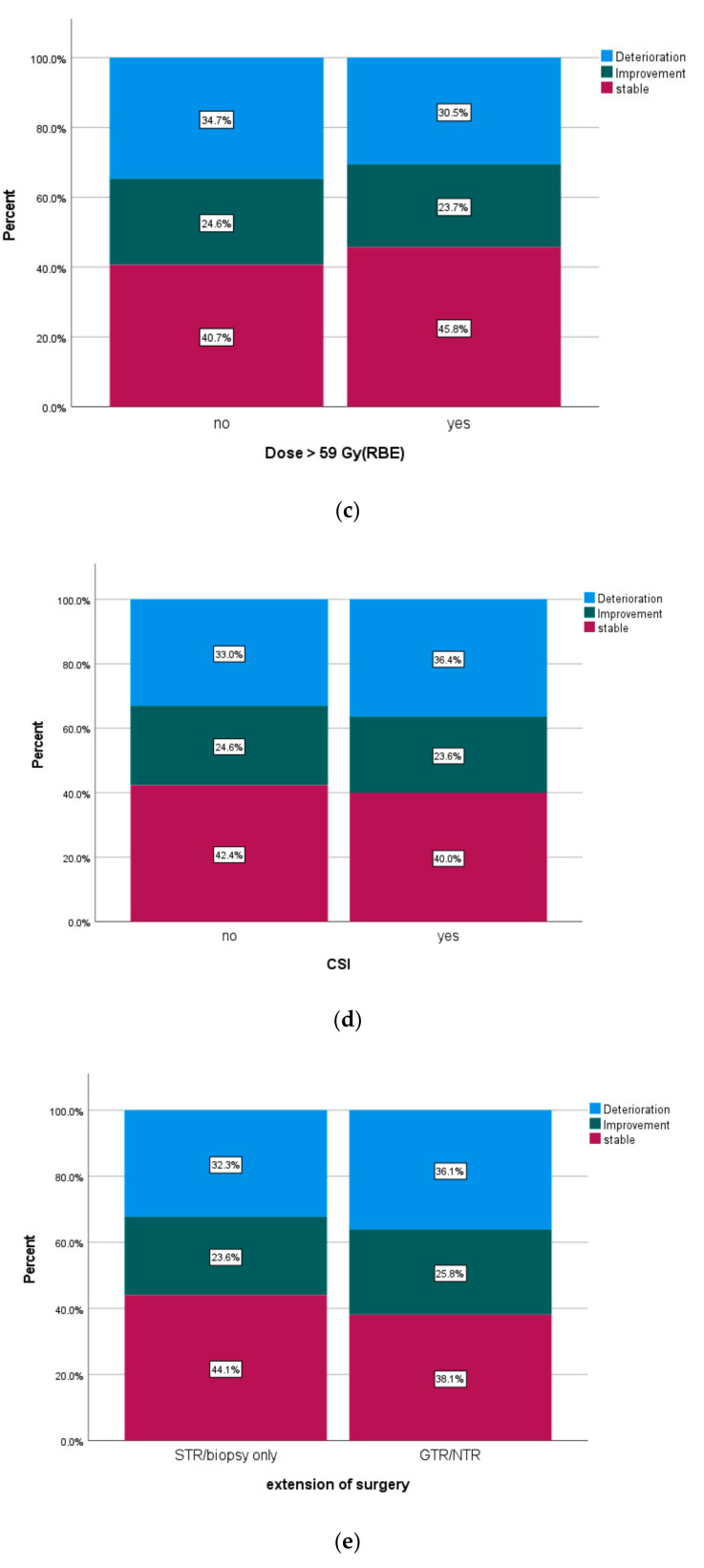

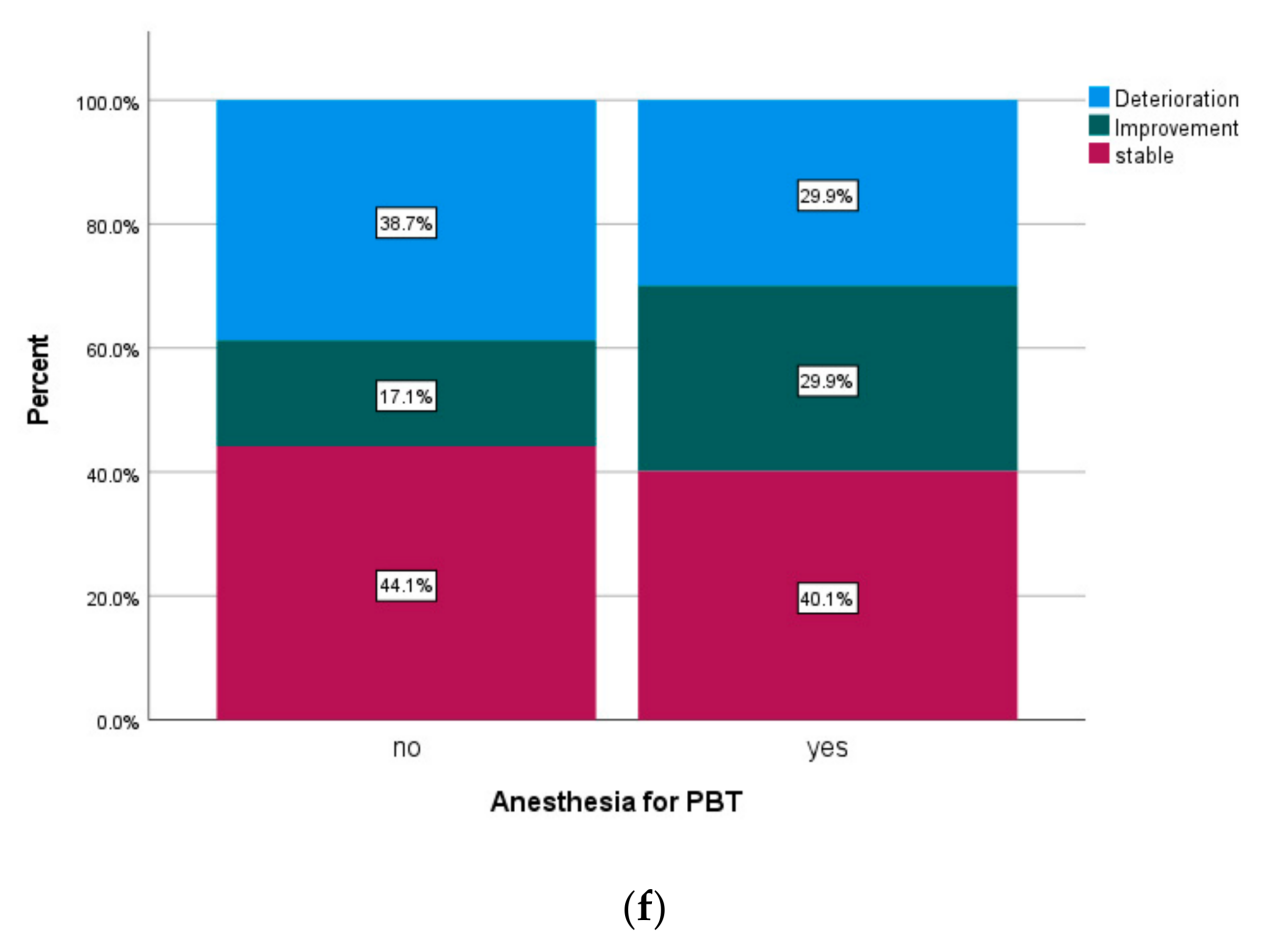
(**a**–**f**) Changes in maximum adverse events of the CNS according to CTCAE Version 4.0 three years after PBT divided by subgroups (**a**) age; (**b**) gender; (**c**) dose; (**d**) CSI; (**e**) extension of surgery; (**f**) anesthesia.

Maximal CTCAE scores for neurotoxicity during FU were available in 258 patients. Compared to the baseline, the last available scores remained stable in 108 patients. Deterioration occurred in 87 and improvement in 63 patients, respectively. Changes in maximum CTCAE scores for different subgroups are displayed in Figure 7a–f.

### 3.4. Review of Image Reports

**Figure 8:**
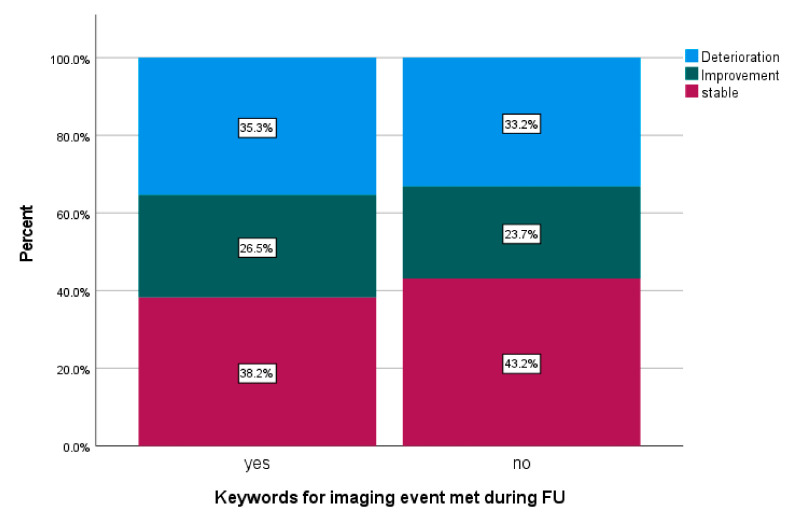
Changes in maximal adverse events of the CNS according to CTCAE Version 4.0 three years after PBT comparing patients with or without imaging events according to predefined keywords.

Written imaging reports were reviewed and met the keyword criteria in 69 patients (23.5%). Tumor location was infratentorial in 42 patients (62.3%). Further patients’ characteristics of this subgroup are displayed in Table A3. Findings occurred after a median FU of 4.3 months (0.47–14.4 months) after PBT. Maximal CTCAE scores for neurotoxicity during FU were available in 68 patients of the subgroups of patients meeting keywords for imaging events and in 190 patients not meeting keywords. Changes of maximal neurotoxicity are displayed in Figure 8.

## 4. Discussion

Within this analysis, we present experiences and follow-up of children with CNS tumors treated with protons from our prospective registry study. Previous studies analyzing a spectrum of pediatric CNS malignancies reported 5-year OS rates between 73.6% and 81.7% [22,23,24]. Therefore, OS found in our cohort of 83.3% was within the expected range. Survival data of the specific entity subgroups was in the range of previously reported outcomes as well [12,25,26,27,28]. In the ependymoma subgroup, PFS was lower than in recently published studies, even when only patients at primary diagnosis are compared. Our cohort presents with a lower age (median 3.1 years) than a previously published cohort, which might be a surrogate for other dismal prognostic factors [29,30]. Three-year OS for patients treated at first diagnosis is in line with other studies [12,28].

ATRT is an aggressive malignancy of the CNS with a dismal prognosis, predominantly in young children [31]. In our analysis, children with an ATRT had lower OS and PFS than other entities in our analysis. This is in line with findings of other groups describing rather poor results of low long-term survival [32,33]. ATRT pathology was also an independent risk factor for outcome in a heterogeneous group of children treated with PBT in a study carried out by Tran et al. [22]. Of note, despite diagnosis of an ATRT in our cohort, one third of children with ATRT did not experience disease progression within three years of FU. 

Interestingly, sedation turned out as a risk factor for all examined outcomes on univariate analysis and remained significant in COX regression when tested for confounding factors. Anesthesia is applied to a predominantly younger cohort, which is more often affected by aggressive entities. It is noteworthy though that age under four years did not remain a significant risk factor in multivariable COX regression. Reasons for the need for anesthesia besides age may be the general treatment burden that prevents a patient from conscious compliance. Anesthesia may therefore be a proxy for a young and more affected subgroup, but further observation is necessary to better understand the underlying causes of our finding. Other studies found age to be a risk factor for disease control and OS in heterogeneous cohorts within univariate analysis, but also did not remain significant in COX regression [22]. Metastatic disease prior to PBT was a significant negative factor after multivariable analysis. Similar results have been presented by Tran et al. [22]. Doses lower than 59 Gy (RBE) were associated significantly with better PFS and LC. However, since this study contained different entities, a lower prescribed dose was also typically given in diagnoses with less aggressive histology.

In general, PBT was well tolerated. Only two patients had to terminate PBT early before achieving the prescribed dose, but none due to acute toxicity. Longer treatment interruptions were all due to malfunction or infection of implanted medical devices and not related to acute treatment toxicity. Schuermann et al. also presented low rates of acute treatment toxicity also considering concomitant chemotherapy in an overlapping cohort [34]. Previous publications on the KiProReg study regarding infants also found PBT to be a part of a complex multimodal treatment, but not being the main reason for acute toxicity and treatment interruptions [14].

Regarding long-term toxicity, incidences of up to 10% of hearing loss after PBT in pediatric patients with CNS tumors were reported in literature [22,30]. Our study revealed results within that range. Two children with supratentorial ATRT showed vision loss after PBT. In one case, the child had been too young to actively participate in ophthalmologic testing prior to PBT, preventing us from knowing the baseline before RT. In those two patients, tumors were in close proximity to the chiasm and the optical nerves. Recent studies on PBT for chordoma did not report vision loss despite high PBT doses unless a Dmax of above 60 Gy (RBE) was applied [35,36]. In both of our cases with vision loss, intrathecal CTX with methotrexate had been administered prior to PBT, demonstrating that especially in ATRT patients the extensive prior treatment needs to be considered when tailoring RT treatment. However, in the majority of patients, scores for neurotoxicity remained stable or even improved during the examined timespan. The most frequent new high-grade neurotoxicity was memory impairment. Even if this is a well-described issue, incidences and methods of reporting differ within the literature [37,38,39,40]. Latest reviews suggest low incidences after focal PBT [41]. Since our cohort had a low median age and a relatively short follow-up observation, this outcome was difficult to report. This could maybe have resulted in an underestimation of this adverse event.

The number of suspicious cases after screening for keywords for imaging events compares with literature describing imaging findings in up to 43% of their cases after PBT [42]. However, the clinical significance of imaging changes after RT is still a matter of discussion; some reports even suggest beneficial long-term outcomes [42]. Definitions used and intracranial sides examined differ among studies, making a comparison more difficult [43,44,45]. The study of Kralik et al. also examined all intracranial tumor locations and could not find a significantly increased risk for infratentorial tumors [18]. Due to the possible prognostic implications, we conducted the retrospective review using the keyword criteria. Interestingly, regarding development of neurological conditions, we did not find any significant difference when compared to the cohort not experiencing any imaging events, suggesting that the majority of imaging events was not associated with any relevant symptoms. In order to further analyze the impact of transient imaging changes and risk factors, centralized review of all imaging studies by an experienced neuroradiologist is ongoing and will serve as the basis for further evaluation.

Our study has several limitations, including the small size of some entity subgroups and the limited observation time. Treatment strategies differed among patients. Standardized systematic neuropsychological testing would be desirable to quantify cognitive outcomes but has not been carried out to date in all patients. Detailed review of endocrinology parameters is ongoing and will be published separately. MRI and reporting available for our cohort were not standardized and quality differed among referring hospitals. 

## 5. Conclusions

The results of our study suggest promising outcomes after PBT. Feasibility and longterm side effects are within the expected ranges. Longer follow-up and larger cohorts are desirable to further assess long-term survival. Standardized neurocognitive testing is needed to investigate cognitive outcomes. Further improvement of treatment strategies is necessary in order to secure high quality of life without compromising survival. Future research may help to identify subgroups of patients with increased risks for severe side effects.

## Figures and Tables

**Table 1:** Keywords for the screening of written reports to analyze imaging events.

Keywords
Radiation necrosis
Imaging changes
White matter lesions
Radiogenic change
Conspicuous, increased T2 intensity/signal
Contrast image (outside tumor remnant)
Gadolinium uptake (outside tumor remnant)
Hemorrhage
Signs of bleeding
Edema
Diffusion disturbance
Barrier disturbance

**Table 2:** Patient characteristics and treatment data (*n* = 294).

Characteristics	*n* (%/Range)
gender	male 172 (58.5%)/female 122 (41.5%)
age at diagnosis	median 4.3 years (0.0–17.7)
age at start of proton therapy	median 5.8 years (0.9–18.0)
WHO grade	
I	44 (15.0%)
II	30 (10.2%)
III	84 (28.6%)
IV	103 (35.0%)
not available/not applicable	33 (11.2%)
resection status	
GTR/NTR	111 (37.8%)
STR	133 (45.2%)
none/biopsy only	50 (17.0%)
localization	
supratentorial	125 (45.8%)
infratentorial	159 (54.2%)
timing of PBT	
primary diagnosis	202 (68.7%)
recurrence/progression	92 (31.3%)
treatment prior to PBT	
prior radiotherapy in an overlapping area	18 (6.1%)
prior chemotherapy	159 (54.1%)
number of tumor surgeries prior to PBT	median 1 (0–7)
treatment during PBT	
concomitant CTX	76 (25.9%)
inpatient treatment besides CTX	51 (17.3%)
time from diagnosis to PBT start	5.4 months (0.8–136.0)
anesthesia during PBT	177 (60.2%)
intraventricular catheter system during PBT	54 (18.4%)
VP/VA shunt during PBT	68 (23.1%)
PBT	
PBS	163 (55.4%)
US	102 (34.7%)
PBS and US	29 (9.9%)
median dose	median 54 Gy (24.0–74.0)
number of fractions	median 30 (6–72)
CSI	70 (23.8%)
interruption of treatment >2 days	22 (7.5%)
time from PBT start to PBT end	median 43 days (11–78)
aftercare	
CTX after PBT	134 (45.9%)
follow-up since first diagnosis	49.2 months (7.2–185.3)
follow-up since end of PBT	median 38.1 months (0.7–83.3)

Abbreviations: PBT = proton beam therapy; GTR = gross total resection; NTR = near total resection; STR = subtotal resection; PBS = pencil beam scanning; US = uniform scanning; CTX = chemotherapy; VA= ventriculoatrial; VP = ventriculoperitoneal; CSI = craniospinal irradiation.

**Table 3:** Univariate analysis of risk factors for OS, PFS, and LC.

Variable	3-Year OS	*p*-Value	3-Year PFS	*p*-Value	3-Year LC	*p*-Value
gender						
female	85.5%		68.7%		80.1%	
male	80.8%	0.233	66.4%	0.549	79.0%	0.939
age ≥ 4 years						
yes	85.7%		75.1%		84.2%	
no	77.4%	0.067	53.8%	0.001	70.8%	0.009
metastatic disease						
yes	70.6%		57.7%		74.1%	
no	84.7%	0.005	68.9%	0.055	80.3%	0.392
time point of PBT						
primary diagnosis	85.6%		68.8%		72.2%	
recurrence/progression	76.2%	0.238	65.6%	0.530	82.2%	0.081
status of resection						
GTR/NTR	87.8%		68.5%		81.3%	
STR/biopsy only	79.5%	0.126	66.9%	0.610	78.4%	0.432
PBT dose						
≥59 Gy (RBE)	76.6%		57.3%		67.4%	
<59 Gy (RBE)	82.8%	0.344	70.1%	0.070	83.2%	0.002
CSI						
yes	79.7%		73.4%		85.6%	
no	83.8%	0.186	65.6%	0.599	77.8%	0.335
anesthesia						
yes	76.9%		57.6%		72.6%	
no	91.7%	0.001	82.5%	<0.001	89.7%	<0.001
concomitant CTX						
yes	80.3%		76.8%		88.4%	
no	83.5%	0.750	63.9%	0.148	76.3%	0.115

Abbreviations: OS = overall survival; PFS = progression-free survival; LC = local control; PBT = proton beam therapy; RBE = relative biological effectiveness; GTR = gross total resection; NTR = near total resection; STR = subtotal resection; CTX = chemotherapy; CSI = craniospinal irradiation.

**Table 4:** Multivariable analysis of risk factors for OS, PFS, and LC.

**Risk factors for OS**	***p*-value**	**HR**	**95% confidence** **interval of HR**
metastatic disease	0.013	2.214	1.181–4.153
status of resection	0.027	1.961	1.079–3.564
anesthesia during PBT	<0.001	0.271	0.135–0.545
**Risk factors for PFS**	***p*-value**	**HR**	**95% confidence** **interval of HR**
PBT dose ≥ 59 Gy (RBE)	0.018	0.578	0.367–0.912
anesthesia during PBT	<0.001	0.530	0.177–0.487
**Risk factors for LC**	***p*-value**	**HR**	**95% confidence** **interval of HR**
PBT dose ≥ 59 Gy (RBE)	0.001	0.414	0.245–0.702
anesthesia during PBT	<0.001	0.334	0.180–0.617
concomitant CTX	0.028	2.063	1.082–3.931

Abbreviations: OS = overall survival; PFS = progression-free survival; LC = local control; HR = hazard ratio; PBT = proton beam therapy; RBE = relative biological effectiveness; CTX = chemotherapy.

## Data Availability

Data available on request due to privacy and ethical restrictions (access date 1 May 2021).

## Appendix A

**Table A1:** Histological entities of included patients.

Histological Entities	*n*	%
Ependymoma	85	28.9
Medulloblastoma	58	19.7
Atypical teratoid rhabdoid tumors	35	11.9
Low-grade glioma	30	10.2
Craniopharyngioma	26	8.8
High-grade glioma	16	5.4
Germ cell tumor intracranial	15	5.1
Retinoblastoma	9	3.1
Choroid plexus tumor	4	1.4
Meningioma	3	1
Neuroblastoma	3	1
Pineoblastoma	3	1
Embryonal tumor with multilayered rosettes	2	0.7
Hemangiopericytoma	1	0.3
Germ cell tumor extracranial	1	0.3
Neurocytoma	1	0.3
Paraganglioma	1	0.3
Rosette-forming glioneuronal tumor	1	0.3

**Table A2:** Patient characteristics and treatment data of subgroup entities.

Characteristics/Entity	Ependymoma	Low-Grade Glioma	ATRT	Medulloblastoma	Cranio-Pharyngioma	Intracranial Germ Cell Tumor	Retinoblastoma	Others
gender	male 36 (42.4%)/female 49 (57.6%)	male 15 (50.0%)/female 15 (50.0%)	male 18 (51.4%)/female 17 (48.6%)	male 41 (70.7%)/female 17 (29.3%)	male 11 (42.3%)/female 15 (57.7%)	male 11 (68.7%)/female 5 (31.3%)	male 8 (88.9%)/female 1 (11.1%)	male 14 (50.0%)/female 14 (50.0%)
age at diagnosis	median 2.4 years (range 0.1–16.9)	median 7.95 (0.2–17.0)	median 1.5 years (range 0.0–7.5 y.)	median 6.2 years (range 0.9–16.2 y.)	median 9.35 years (range 2.5–15.5 y.)	median 11.1 years (range 2.9–16.3 y.)	median 1.0 years (range 0–3.7 y.)	median 5.95 years (range 1.8–17.7 y.)
age at start of PBT	median 3.1 years (range 0.9–17.0)	median 11.4 (4.6–17.9)	median 1.9 years (range 1.0–8.0 y.)	median 6.5 years (range 2.0–16.5 y.)	median 10.65 years (range 5.4–17.1 y.)	median 11.5 years (range 3.3–16.6 y.)	median 2.5 years (range 1.2–8.0 y.)	median 9.0 years (range 2.1–18.0 y.)
resection status								
GTR/NTR	50 (58.8%)	0	10 (28.6%)	34 (58.6%)	0	6 (37.5%)	0	12 (42.8%)
STR	34 (40%)	14 (46.7%)	24 (68.6%)	20 (34.5%)	22 (84.6%)	2 (12.5%)	0	11 (39.3%)
none/biopsy only	1 (1.2%)	16 (53.3%)	1 (2.9%)	4 (6.9%)	4 (15.4%)	8 (50%)	9 (100%)	5 (17.9)
localization								
supratentorial	15 (17.6%)	25 (83.3%)	16 (45.7%)	0	26 (100%)	16 (100%)	9 (100%)	21 (75%)
infratentorial	70 (82.4%)	5 (16.7%)	19 (54.3%)	58 (100%)	0	0	0	7 (25%)
number of surgeries prior to PBT	median 2 (1–6)	median 1 (0–4)	median 1 (0–7)	median 1 (1–3)	median 2 (1–7)	median 1 (0–3)	0	median 1 (0–3)
metastasis prior to PBT	5 (5.9%)	4 (133%)	6 (17.1%)	18 (31.0%)	0	2 (12.5%)	1 (11.1%)	5 (17.9%)
timepoint of PBT								
primary diagnosis	68 (80%)	12 (40.0%)	33 (94.3%)	47 (81.0%)	3 (11.5%)	15 (93.8%)	4 (44.4%)	13 (46.4%)
recurrence/progression	17 (20%)	18 (60.0%)	2 (5.7%)	11 (19.0%)	23 (88.5%)	1 (6.7%)	5 (55.6%)	15 (53.6%)
reservoir during PBT	7 (8.2%)	3 (10%)	23 (65.7%)	12 (20.7%)	6 (23.1%)	2 (12.5%)	0	3 (10.7%)
VP/VA shunt during PBT	28 (32.9%)	6 (20.0%)	12 (34.3%)	13 (22.4%)	5 (19.2%)	0	0	2 (7.1%)
prior RT in overlapping area	7 (8.2%)	0	1 (2.9%)	4 (6.9%)	1 (3.8%)	0	4 (44.4%)	1 (3.6%)
prior chemotherapy	41 (48.2%)	16 (53.3%)	35 (100%)	30 (51.7%)	0	16 (100%)	8 (88.9%)	14 (50.0%)
concomitant CTX	10 (11.8%)	1 (3.3%)	15 (42.9%)	55 (60.3%)	0	0	0	9 (32.1%)
inpatient treatment besides CTX	15 (17.6%)	4 (13.3%)	13 (37.1%)	15(19.5%)	2(7.7%)	0	2 (22.2%)	3 (10.7%)
anesthesia during PBT	69 (81.2%)	23 (76.7%)	34 (97.1%)	37 (63.8%)	3 (11.5%)	1 (6.3%)	9 (100%)	14 (50.0%)
PBT technique								
PBS	35 (41.2%)	13 (5.7%)	17 (46.6%)	38 (65.5%)	18 (59.2%)	13 (81.3%)	4 (44.4%)	20 (71.4%)
US	45 (52.9%)	16 (45.7%)	16 (45.7%)	3 (5.2%)	8 (30.2%)	3 (18.7%)	5 (55.6%)	4 (14.3%)
PBS and US	5 (5.9%)	1 (2.7%)	2 (5.7%)	17 (29.3%)	0	0	0	4 (14.3%)
median dose	median 54.04 (range 49.6–62.0 Gy)	median 54.0 (range 46.0–554 Gy)	median 54.0 Gy (range 36.6–60.0 Gy)	median 54.0 Gy (range 30.0–72.0 Gy)	54.0 Gy	54.0 Gy (24–54 Gy)	50.0 Gy (49,6–50,0 Gy)	54.0 Gy (45–72,0 Gy)
number of fractions	median 31 (range 30–33)	median 30 (28–33)	median 30 (range 22–35)	median 30 (range 6–72)	30	30 (15–30)	30 (15–30)	31 (25–70)
CSI	2 (2.4%)	1 (3.3%)	6 (17.1%)	50 (86.2%)	0	2 (12.5%)	1 (11.1%)	8 (28.6%)
chemotherapy after PBT	32 (37.9%)	9 (24.3%)	13 (37.1%)	47 (81.0%)	0	2 (12.5%)	3 (33.3%)	13 (46.4%)
interruption of PBT treatment > 2 days	10 (11.8%)	2 (6.7%)	4 (11.4%)	2 (3.4%)	2 (7.7%)	1 (6.3%)	1 (11.1%)	2 (7.1%)
Follow-up since end of PBT	median 39.7 months (3.2–83.3 m)	median 49.95 months (7.7–76.7 m)	median 34.2 months (range 0.7–76.1 m)	median 36.0 months (range 2.7–67.1 m)	median 47.9 months (range 11.7–77.5 m)	median 35.5 months (range 20.3–81.7 m)	median 38.2 months (range 3.0–52.9 m)	median 34.1 months (range 3.9–73.3 m)

Abbreviations: PBT = proton beam therapy; GTR = gross total resection; NTR = near total resection; STR = subtotal resection; PBS = pencil beam scanning; US = uniform scanning; CTX = chemotherapy; VP = ventriculoperitoneal; VA = ventriculoatrial; CSI = craniospinal irradiation; ATRT = atypical teratoid rhabdoid tumor.

**Table A3:** Patient characteristics and treatment data of subgroups divided by meeting the criteria for imaging events.

Characteristic	Patients Not Meeting the Criteria for Imaging Events	Patients Meeting Criteria for Imaging Events
	*n* (%/Range)	*n* (%/Range)
gender	male 131 (58.2%)/female 94 (41.8%)	male 41 (59.4%)/female 28 (40.6%)
age at diagnosis	median 5.0 years (0.0–17.5)	median 3.8 years (0.4–17.7)
age at start of PBT	median 6.3 years (1.0–18.0)	median 4.8 years (0.9–17.9)
resection status		
GTR/NTR	77 (24.2.3%)	34 (49.3%)
STR	103 (45.8%)	30 (43.5%)
none/biopsy only	45 (20.0%)	5 (7.2%)
localization		
supratentorial	109 (48.4%)	26 (37.7%)
infratentorial	116 (51.6%)	43 (62.3%)
timing of PBT		
primary diagnosis	148 (65.8%)	54 (78.3%)
recurrence/progression	77 (34.2%)	15 (21.7%)
treatment prior to PBT		
prior radiotherapy in an overlapping area	14 (6.2%)	4 (5.8%)
prior CTX	120 (53.3%)	39 (56.5%)
prior intrathecal CTX	40 (17.8%)	19 (27.5%)
number of tumor surgeries prior to PBT	median 1 (0–6)	median 1 (0–7)
treatment during PBT		
concomitant chemotherapy	51 (22.7%)	26 (37.7%)
reservoir during PBT	41 (18.2%)	14 (20.3%)
VP/VA shunt during PBT	49 (21.8%)	19 (27.5%)
PBT		
PBS	130 (57.8%)	34 (49.3%)
US	75 (33.3%)	26 (37.7%)
PBS and US	20 (8.9%)	9 (13.0%)
median dose	median 54 Gy (24.0–72.0)	median 54 Gy (49.6–68.0)
number of fractions	median 30 (6.0–72)	median 30 (30–67)
CSI	57 (35.3%)	13 (18.8%)
interruption of treatment > 2 days	17 (7.6%)	4 (5.8%)
aftercare		
CTX after PBT	96 (42.7.1%)	38 (55.1%)
FU since end of PBT	median 36.0 months (0.7–81.7)	median 45.3 months (10.1–83.3)

Abbreviations: PBT = proton beam therapy; GTR = gross total resection; NTR = near total resection; STR = subtotal resection; PBS = pencil beam scanning; US = uniform scanning; CTX = chemotherapy; VP = ventriculoperitoneal; VA = ventriculoatrial; CSI = craniospinal irradiation.
